# Incidence of familial Hodgkin's disease.

**DOI:** 10.1038/bjc.1983.109

**Published:** 1983-05

**Authors:** L. Kerzin-Storrar, M. J. Faed, J. B. MacGillivray, P. G. Smith

## Abstract

The family histories of 131 patients with histologically defined Hodgkin's disease (HD) were studied and 2,517 first and second degree relatives and spouses were identified and followed-up. The causes of death in deceased relatives were ascertained from death certificates. The numbers of deaths from selected causes were compared with the numbers that would be expected if the relatives had suffered the same mortality rates as the Scottish national population. A 4-fold increase in deaths due to HD was found among first and second degree relatives of patients with the disease (6 cases observed compared with 1.4 expected). Five of the 6 familial cases were related to index patients with the mixed cellularity form of the disease, the remaining case was the brother of a patient with the lymphocyte-depleted form of the disease. The increased risk was seen among relatives of both young and older patients and there was no consistent intrafamilial similarity in age of onset or time of onset of disease.


*Br. J. Cancer* (1983), **47**, 707–712

### Incidence of familial Hodgkin's disease

L. Kerzin-Storrar, M.J.W. Faed, J.B. MacGillivray & P.G. Smith[1]

*Department of Pathology, Ninewells Hospital and Medical School, Dundee, Scotland and [1]Department of Medical Statistics and Epidemiology, London School of Hygiene and Tropical Medicine, London.*

**Summary** The family histories of 131 patients with histologically defined Hodgkin's disease (HD) were studied and 2,517 first and second degree relatives and spouses were identified and followed-up. The causes of death in deceased relatives were ascertained from death certificates. The numbers of deaths from selected causes were compared with the numbers that would be expected if the relatives had suffered the same mortality rates as the Scottish national population. A 4-fold increase in deaths due to HD was found among first and second degree relatives of patients with the disease (6 cases observed compared with 1.4 expected). Five of the 6 familial cases were related to index patients with the mixed cellularity form of the disease, the remaining case was the brother of a patient with the lymphocyte-depleted form of the disease. The increased risk was seen among relatives of both young and older patients and there was no consistent intrafamilial similarity in age of onset or time of onset of disease.

The aetiology of Hodgkin's Disease (HD) is unknown although evidence exists to implicate the involvement of both genetic and environmental factors. An increased risk of HD among first degree relatives (Razis *et al.*, 1959) and siblings (Grufferman *et al.*, 1977) of HD patients has been shown. The finding of a significant association between HD and parental consanguinity (Abramson *et al.*, 1978) and the results of statistical analysis of data from a family in which several members had HD (Thompson, 1981) suggests an autosomal recessive HD-susceptibility gene. Case reports of HD occurring in families with known immunodeficiency disorders (McBride & Fenelly, 1977; Buehler *et al.*, 1975; Harris *et al.*, 1981) and the suggested association between HD and certain HLA haplotypes are consistent with the hypothesis that inherited susceptibility to HD may be determined by immune response genes (Marshall *et al.*, 1977; Bowers *et al.*, 1977). Recent epidemiological data suggest that HD, at least in young people, may be a rare sequel to a common virus infection. It has been postulated that a decreased exposure to infections in childhood and a later-than-average age at infection with a common virus may predispose towards the development of the disease (Gutensohn & Cole, 1981).

It is also possible that there is more than one aetiology for the disease depending upon histological type (Lukes *et al.*, 1966) and age at onset (Cole *et al.*, 1968; Gutensohn & Cole, 1977). With these possibilities in mind we have investigated the causes of death of first and second degree relatives and spouses of patients with HD.

Correspondence: M.J.W. Faed, Cytogenetics Laboratory, Department of Pathology, Ninewells Hospital and Medical School, Dundee, DD1 9SY.

Received 6 December 1982; accepted 26 January 1983.

### Materials and methods

### *Index cases*

Two hundred and three cases of HD were identified in the pathology records of the Dundee hospitals for the 31-year period, 1950–1980. Nineteen cases were excluded from the study as pathological material was unavailable. The remaining 184 cases were reviewed by one pathologist (J. McG.) and for 14 the original diagnosis of HD was not confirmed. The remaining 170 cases (97 males and 73 females) were subtyped according to the Rye classification (Lukes *et al.*, 1966).

Pedigrees of first and second degree relatives were completed for 131 (71%) of these 170 patients. The histological subtypes and ages at onset are shown in Table I.

### *Pedigrees*

*Deceased patients* First and second degree relatives and spouses of decreased patients were identified using Scottish Registration records which contain details of all births, marriages, and deaths in Scotland since 1855. Annual indexes are arranged alphabetically by surname for each sex, listing the place of registration and, from 1929 onwards, the mother's maiden name. Sibships were assembled by searching through the birth indexes from the year of the parent's marriage to the year in which the

© The Macmillan Press Ltd., 1983

**Table I** Distribution of index patients according to (a) histologic subtype and (b) age at onset

| (a) Histologic Subtype | Confirmed Cases Total | Number Studied |
|---|---|---|
| Nodular sclerosis | 43 | 33 |
| Mixed cellularity | 79 | 61 |
| Lymphocyte depleted | 25 | 20 |
| Lymphocyte predominant | 18 | 13 |
| Uncertain classification | 5 | 4 |
| TOTAL | 170 | 131 |

| (b) Age at Onset | Confirmed Cases Total | Number Studied |
|---|---|---|
| 0–15 years | 10 | 5 |
| 16–39 years | 76 | 61 |
| ≥40 years | 84 | 65 |
| TOTAL | 170 | 131 |

mother reached the age of 50 years (in the case of divorce or early death offspring of remarriages were sought in the same way). The birth certificates were then examined and identifying information (full name and occupation of parents and date of place of parent's marriage) was checked before including an individual in a pedigree. The parent's marriage certificate included their ages at marriage and their own parent's names so that sibships in previous generations could be compiled. Successive generations were completed after marriages had been identified from annual indexes (searched, for each individual, from the age of 16–45 years).

Of the total of 118 deceased patients, 90 family histories were successfully traced. We were unable to establish pedigrees for 15 patients; 2 of these patients had been adopted and the others were patients from early in the study period when little identifying information was available from hospital records. A further 13 pedigrees were not compiled because of time limitations.

*Living patients* Patients who were alive at the time of the study (or their parents for 3 juvenile cases) were interviewed either at the hospital lymphoma clinic or in their homes. Three patients who had moved to England were sent a questionnaire. Details of all first and second degree relatives and spouses were obtained including their birth dates, occupation and, if deceased, date and cause of death. This information was confirmed and expanded by searching Scottish Registration records (explained above).

Of the 52 living patients, 36 were interviewed and 3 completed questionnaires. Thirteen patients were not interviewed either because the General Practitioner or patient refused permission, or because the patient had moved abroad or to an unknown address. Of the 13, 2 pedigrees were compiled as for deceased patients and the remaining 11 were not included in the study.

To assess the efficacy of the procedures used to ascertain relatives through Scottish Registration records, we compiled 5 pedigrees of living patients using this method, prior to interview. In all instances the pedigrees derived were found to be at least as complete as the family histories given by patients themselves.

*Mortality*

For those relatives whose current status was uncertain an attempt was made to trace them through the National Health Service (NHS) Central Register and the Department of Health and Social Security (DHSS). Annual death indexes for Scotland were searched also. Death certificates were examined for all those relatives who were known, or were found to have died, and the primary cause of death was coded according to the 8th Revision of the International Classification of Disease, Injury and Causes of Death (W.H.O. Manual, 1968).

Expected numbers of deaths among relatives were calculated by multiplying the age, sex, and time specific person years at risk accumulated by the study group by corresponding Scottish national mortality rates. Person years at risk were computed for males and females in 5-year age groups up to the age of 84 years, years at risk after the age of 84 years were considered in one group; and in quinquennial periods from 1911 (the first year for which rates are given by Case *et al.* (1976)) up until 1981, the end of the study period (the Scottish national mortality rates for 1976–1981 were not readily available to us and in the calculations those for 1970–1975 were used for this period). Person years at risk were computed as follows: For any siblings, children, grandchildren, aunts, uncles, nieces and nephews of a patient, the start of the period of risk was taken as their date of birth; for the mother of a patient the start was taken as the patient's date of birth and for the father as the estimated date of conception (9 months before the patient's date of birth); for grandmothers the risk period was started at the date of birth of the patient's mother (or father) and for grandfathers it was taken to be 9 months before this date; for spouses the risk period was started at the date of marriage. Person years at risk prior to 1911 were ignored in the analysis as were deaths occurring before that year.

Person years at risk were accumulated for each individual up to date of death or emigration from the United Kingsom, or in the case of living individuals the date within the study period he or she was last confirmed to be alive.

Differences between the observed and expected numbers of deaths were tested for statistical significance by assuming that the actual number of deaths was sampled from a Poisson distribution with mean equal to the expected number of deaths. The significance tests shown are one-sided in the direction of the observed difference.

### Results

Three thousand, one hundred and twenty-eight relatives were identified (Table II). Of these, 363 died before 1911 and have been excluded from further analysis. A further 248 could not be followed-up. Of the remainder, 1480 were still alive at the time of the study, 999 had died, 22 had emigrated from the United Kingdom, and 16 were lost-to-follow-up.

Table III shows the number of observed and expected deaths among relatives and spouses of the index patients. The overall mortality rate was 86% of that of the Scottish national death rate over the study period. This deficit of deaths (999 deaths against 1167.2 expected) was statistically highly significant. The number of deaths from neoplasms was very close to expected (186 observed against 186.1 expected) and the deficiency was due to deaths from non-neoplastic conditions (813 against 981.1; ratio = 0.83).

There were 6 deaths from HD while 1.4 were expected. This 4-fold excess of deaths due to HD was highly significant ($P<0.01$). Two of the index cases were related and they comprise 2/6 deaths from HD among relatives. The relationship between these patients was not known to us before the study was started. As the 2 cases were ascertained independently each was counted in the analysis and a pedigree for each of the 2 cases was constructed. Both pedigrees were included in the computation of person years at risk and hence in the calculation of the expected number of cases. There were no deaths

**Table II** Ascertainment and follow-up of relatives of index patients

| | *First Degree** | *Second Degree** | *Spouses* | *Total* |
|---|---|---|---|---|
| Total ascertained | 811 | 2217 | 100 | 3128 |
| Death pre 1911 | 36 | 327 | 0 | 363 |
| Unable to be followed-up | 17 | 230 | 1 | 248 |
| Total excluded | 53 | 557 | 1 | 611 |
| Alive | 483 | 923 | 74 | 1480 |
| Dead | 264 | 711 | 24 | 999 |
| Emigrated | 4 | 17 | 1 | 22 |
| Lost to follow-up | 7 | 9 | 0 | 16 |
| Total included in analysis | 758 | 1660 | 99 | 2517 |

*The definition of first and second degree relatives is based on the proportion of genes shared in common. First degree relatives include parents, siblings and children. Second degree relatives include grandparents, aunts and uncles, half-siblings, nieces and nephews and grandchildren.

**Table III** Observed and expected deaths by cause in relatives of index patients

| | *First and second degree relatives* | | | *Spouses* | | | *Total* | | |
|---|---|---|---|---|---|---|---|---|---|
| | *Observed* | *Expected* | *O/E* | *Observed* | *Expected* | *O/E* | *Observed* | *Expected* | *O/E* |
| All causes | 975 | 1136.35 | 0.86† | 24 | 30.90 | 0.78 | 999 | 1167.25 | 0.86† |
| All neoplasms | 179 | 179.80 | 1.00 | 7 | 6.32 | 1.11 | 186 | 186.11 | 1.0 |
| Hodgkin's disease | 6 | 1.37 | 4.38* | 0 | 0.06 | 0 | 6 | 1.43 | 4.20* |
| Leukaemia | 3 | 3.38 | 0.89 | 0 | 0.13 | 0 | 3 | 3.51 | 0.85 |
| Causes other than neoplasms | 796 | 956.56 | 0.83† | 17 | 24.58 | 0.69 | 813 | 981.10 | 0.83† |

*$P<0.01$.
†$P<0.001$.

from HD among the spouses but only 0.06 were expected.

There were 3 deaths from leukaemia and 3.5 were expected. In addition there were 2 deaths from lymphomas other than HD but expected numbers have not been calculated as national rates were not available for this cause for the total study period.

Five of the 6 familial cases of HD occurred among relatives of index patients whose disease was classified histologically as mixed cellularity while 0.7 were expected (Table IV). The remaining case was a relative of an index case with the lymphocyte depleted form of HD.

Three of the familial cases of HD were relatives of index patients whose disease onset was at age <45 years and three were related to index cases with disease onset at age ⩾45 years (Table IV). The excess risk was statistically significant for both age groups.

Four of the observed cases of HD were first degree male relatives, one was a second degree male relative and one was a second degree female relative. All of the index cases with affected relatives were male (Tables IV and V).

### Discussion

This study found a 4-fold increased risk of death from HD among the first and second degree relatives of HD patients. For various reasons it was not possible to construct pedigrees for 39 (23%) of the 170 index patients and 264 (8%) of the relatives of the 131 index patients included were lost to follow-up. We have no reason to suppose that the persons who were successfully traced were a biased subset with respect to their risk of HD but even if we assume that there were no deaths from HD among the relatives of untraced index patients and assume all relatives were completely followed-up the expected number of deaths would still be below 2.0, whereas 6 deaths were observed.

Previous reports of an increased familial incidence of HD have been limited to surveys of close relatives—either siblings (Grufferman *et al.*, 1977) or first degree relatives (Razis *et al.*, 1959)—of cases. In this study we have included all first and second degree relatives of patients and, overall,

**Table IV** Observed and expected deaths due to HD in relatives, according to (a) histologic subtype of index case, (b) age at onset of index case and (c) first or second degree relative

| | *Observed* | *Expected* | *O/E* |
|---|---|---|---|
| (a) | | | |
| Nodular sclerosis | 0 | 0.35 | 0 |
| Mixed cellularity | 5 | 0.66 | 7.6‡ |
| Lymphocyte depleted | 1 | 0.22 | 4.5 |
| Lymphocyte predominant | 0 | 0.10 | 0 |
| Uncertain classification | 0 | 0.04 | 0 |
| (b) | | | |
| Onset <45 years | 3 | 0.75 | 4.0* |
| Onset ≥45 years | 3 | 0.62 | 4.8* |
| (c) | | | |
| First degree relatives | 4 | 0.44 | 9.1† |
| Second degree relatives | 2 | 0.93 | 2.2 |
| First and second degree relatives | 6 | 1.37 | 4.40† |

*$P<0.05$.
†$P<0.01$.
‡$P<0.001$.

**Table V** Familial cases of HD

| | *Index case* | | | | *Affected relative* | | | | |
|---|---|---|---|---|---|---|---|---|---|
| | *Sex* | *Histological type** | *Age at onset* | *Year of onset* | *Sex* | *Relation to index patient* | *Histological type** | *Age at death* | *Year of death* |
| 1† | M | MC | 25 | 1965 | M | father | MC | 49 | 1968 |
| 2† | M | MC | 48 | 1964 | M | son | MC | 27 | 1967 |
| 3 | M | MC | 74 | 1963 | M | father | not known | 59 | 1911 |
| 4 | M | MC | 22 | 1963 | M | paternal grandfather | not known | 25 | 1915 |
| 5 | M | MC | 50 | 1963 | F | paternal aunt | not known | 69 | 1963 |
| 6 | M | LD | 25 | 1954 | M | brother | MC | 26 | 1952 |

*MC—mixed cellularity; LD—lymphocyte depleted.
†Index cases 1 and 2 are father and son.

have found a 4-fold excess of cases of HD, though the excess is most marked among first degree relatives (Table IV).

It has been suggested that HD may be 2 separate diseases and that the form affecting young people is aetiologically distinct from that found in older patients (Gutensohn & Cole, 1977; 1981; Cole *et al.*, 1968). In the study of Grufferman *et al.* (1977) the excess risk among siblings was confined to those with disease onset below 45 years of age. The excess among relatives of HD patients found in our study occurred within families of patients with both young onset and late onset disease suggesting that familial factors influence the development of HD regardless of age of the patient.

Previous reports of multiply affected HD families have included cases in all of the four histological subtypes, though there has been some suggestion that nodular sclerosing HD is the most common familial type (Hull & Delamore, 1978). In our study there were only 6 cases of HD among relatives but it is notable that none of these cases occurred in relatives of patients with the nodular sclerosing form of disease. Unfortunately, we do not know the histological subtype of 3/6 cases among relatives (Table V). The father-son pair of cases both had disease of the mixed cellularity type. There were 5 cases in relatives of patients with the mixed cellularity form of disease and one occurred in a relative of an index case with the lymphocyte depleted form. The relative, however, had the mixed cellularity form of the disease.

The pedigrees do not suggest a simple Mendelian pattern of inheritance. In 2 of the families the time interval between cases (for index patients date of onset and, for relatives, date of death) was much greater than the difference in ages at occurrence (or death), while in another 2 families the reverse was true. In the fifth family 2 brothers were diagnosed at the same age within 2 years of one another. We calculated the observed and expected number of cases occurring within 3 years of each other, 3–9 years, 9–15 years or more than 15 years (i.e. time between onset of the index case and death of the relative). For each of these time periods observed and expected numbers were, respectively, 3, 0.17; 1, 0.30; 0, 0.26, and 2, 0.70. Thus there was some evidence that the excess was greatest for pairs of persons developing the disease at about the same time. A similar comparison was made with respect to *age* at developing the disease (i.e. age at onset of the index case and age at death of the relative) and the corresponding observed and expected numbers were 2, 0.11 (i.e. cases with age at onset and age at death within 3 years of each other); 0, 0.22; 1, 0.21 and 3, 0.89 (ages more than 15 years apart). Again there was some suggestion that the excess was greatest for pairs of persons developing the disease at about the same age. It should be noted, however, that these comparisons are based upon small numbers of cases and the available data are not sufficient to distinguish between shared genes or environment as the cause of the increased incidence.

An association between familial history of cancer and HD was found in a large retrospective study comparing men who developed HD with their classmates at college who did not develop the disease (Paffenbarger *et al.*, 1978). In the present study there was no overall increased risk of death due to neoplasms in relatives of patients with HD.

The deficit of deaths from causes other than neoplasms (813 observed, 981.1 expected) may be due to underascertainment of deaths in our study group. This could have arisen if persons in the study had died abroad or the death had not been notified back to the NHS Central Register or to the DHSS. If this is the explanation there is no reason to suppose that deaths from cancer would not have been similarly underascertained. In which case our finding of 186 deaths from neoplasms against 186.1 expected might indicate a true excess of deaths from cancer. This is rather a speculative suggestion, however, as we do not know what proportion of deaths, if any, we have failed to detect. Also it is possible that the relatives of patients with Hodgkin's disease are at decreased risk of death from some non-neoplastic causes. Patients with Hodgkin's disease tend to be of higher than average social class (Guttensohn & Cole, 1981) and thus their relatives might be expected to have lower than expected mortality—though it is unlikely that this can explain all of the deficit. We have not examined in detail individual causes of death in the non-neoplastic group (as mortality rates for non-neoplastic causes are not published in summary form from 1911 as are cancer mortality rates) but the number of deaths from tuberculosis (31 against 38.0 expected) and bronchopneumonia (43 against 39.1 expected) were close to expectation.

In conclusion, this study documents a 4-fold increase in risk of HD to first and second degree relatives of affected individuals. Five of the 6 familial cases were related to index cases with the mixed cellularity form of HD, and the relative of the one case with the lymphocyte depleted HD had the mixed cellularity type. The increased risk is seen among families of both young and older onset patients and there is no consistent intrafamilial similarity in age of onset. No obvious common exposure to an environmental hazard at particular times is indicated as some of the dates of onset in the familial cases were widely separated in time. The number of spouses in the study was too small to assess risk or for useful comments on the possibility of exposure to a common relevant environmental hazard during adult life. Although finding 6/131 cases of HD to have an affected first or second degree relative does not suggest a strong genetic effect in HD, the distribution of cases within families would be consistent with the inheritance of a susceptibility gene, perhaps operating as an inherited abnormality of the immune response. However, the possibility that the observed excess in families is due to common environmental exposures cannot be ruled out.

We thank Drs. G.R. Tudhope and J.S. Scott for their co-operation and permission to interview patients. We are grateful for help provided by the DHSS and the NHS Central Register of the General Register Office for Scotland and to Ms. D. Carson and Ms. K. Barratt for computing assistance and to Ms. D. Morrison and Ms. J. Partridge for clerical help. The study was funded by a grant from the Leukaemia Research Fund.

### References

ABRAMSON, J.H., PRIDAN, H., SACKS, M.I., AVITZOUR, M. & PERITZ, E.A. (1978). A case-control study of Hodgkin's disease in Israel. *J. Natl Cancer Inst.*, **61**, 307.

BOWERS, T.K., MOLDOW, C.F., BLOOMFIELD, C.D. & YUNIS, E.J. (1977). Familial Hodgkin's disease and the major histocompatibility complex. *Vox Sang.*, **33**, 273.

BUEHLER, S.K., FIRME, F., FODOR, G., FRASER, G.R., MARSHALL, W.H. & VAZE, F. (1975). Common variable immunodeficiency, Hodgkin's disease and other malignancies in a Newfoundland family. *Lancet*, **i**, 195.

CASE, R.A.M., COGHILL, D., DAVIES, J.M. & 6 others. (1976). *Serial Mortality Tables: Neoplastic Diseases, 4, Scotland 1911–1970.* London: Division of Epidemiology, Institute of Cancer Research.

COLE, P., MCMAHON, B. & AISENBERG, A. (1968). Mortality from Hodgkin's disease in the United States. Evidence for the multiple-aetiology hypothesis. *Lancet*, **ii**, 1371.

GRUFFERMAN, S., COLE, P., SMITH, P.G. & LUKES, R.J. (1977). Hodgkin's disease in siblings. *N. Engl. J. Med.*, **296**, 248.

GUTENSOHN, N. & COLE, P. (1981). Childhood social environment and Hodgkin's disease. *N. Engl. J. Med.*, **304**, 135.

GUTENSOHN, N. & COLE, P. (1977). Epidemiology of Hodgkin's disease in the young. *Int. J. Cancer*, **19**, 595.

HARRIS, R.E., BAEHNER, R.L., GLEISER, S., WEAVER, D.D. & HODES, M.E. (1981). Cartilage-hair hypoplasia, defective T-cell function and Diamond-Blackfan anaemia in an Amish child. *Am. J. Med. Genet.*, **8**, 291.

HULL, P.J. & DELAMORE, I.W. (1978). Familial Hodgkin's disease. *Postgrad. Med. J.*, **54**, 676.

LUKES, R.J., CRAVER, L.F., HALL, T.C., RAPPAPORT, H. & RUBEN, P. (1966). Report of the nomenclature committee. *Cancer Res.*, **26**, 1311.

MARSHALL, W.H., BARNARD, J.M., BUEHLER, S.K., CRUMLEY, J. & LARSEN, B. (1977). HLA in famililal Hodgkin's disease. Results and a new hypothesis. *Int. J. Cancer*, **19**, 450.

MCBRIDE, A. & FENELLY, J. (1977). Immunological depletion contributing to familial Hodgkin's disease. *Eur. J. Cancer*, **13**, 549.

PAFFENBARGER, R.S., WING, A.L. & HYDE, R.T. (1978). Characteristics in youth predictive of adult-onset malignant lymphomas, melanomas and leukaemias. *J. Natl Cancer Inst.*, **60**, 89.

RAZIS, D.V., DIAMOND, H.D. & CRAVER, L.F. (1959). Familial Hodgkin's disease: its significance and implications. *Ann. Intern. Med.*, **51**, 933.

THOMPSON, E.A. (1981). Pedigree analysis of Hodgkin's disease in a Newfoundland genealogy. *Ann. Hum. Genet.*, **45**, 279.

WORLD HEALTH ORGANISATION (1968). *Manual of the International Statistical Classification of Disease, Injuries, and Causes of Death.* Eighth revision, Geneva: World Health Organisation.


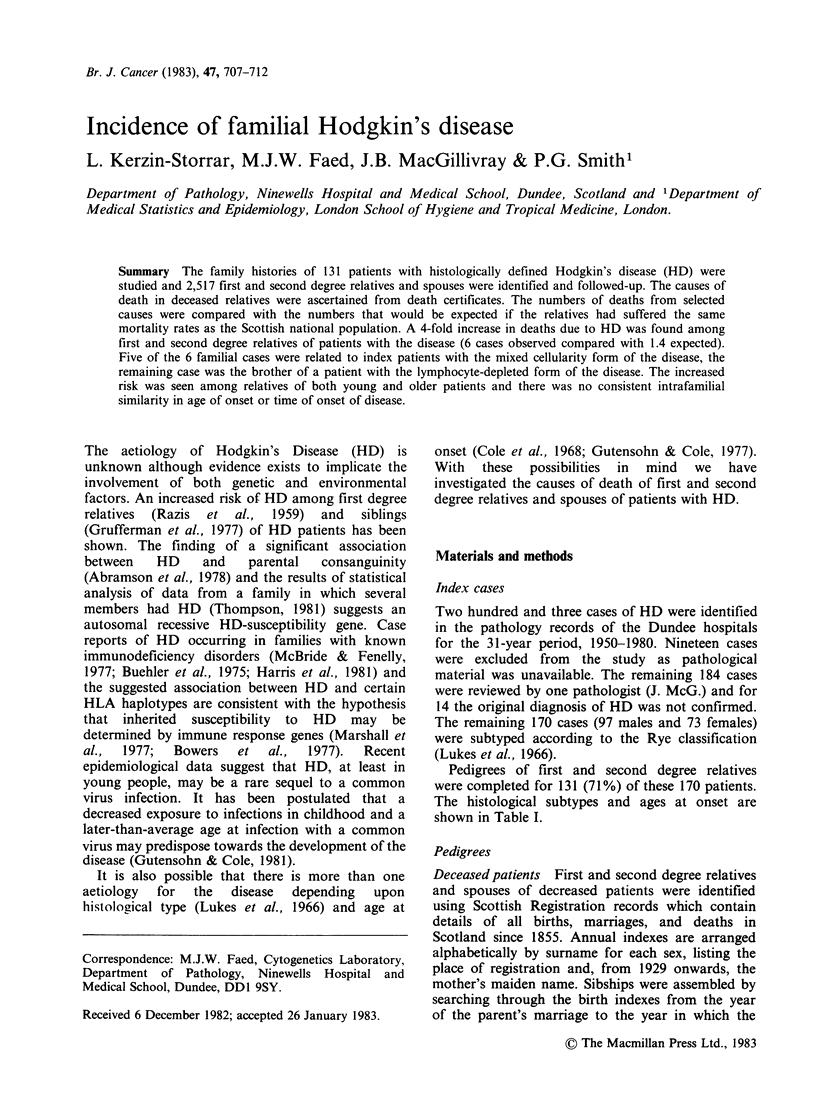

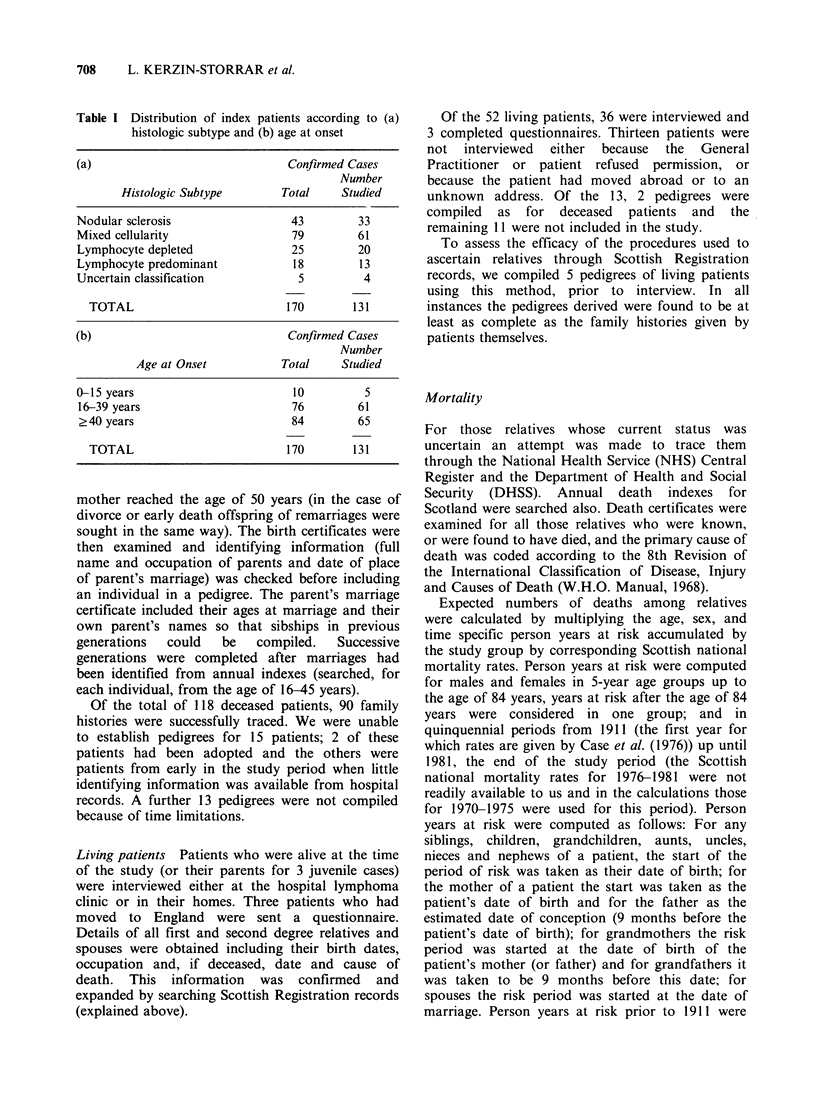

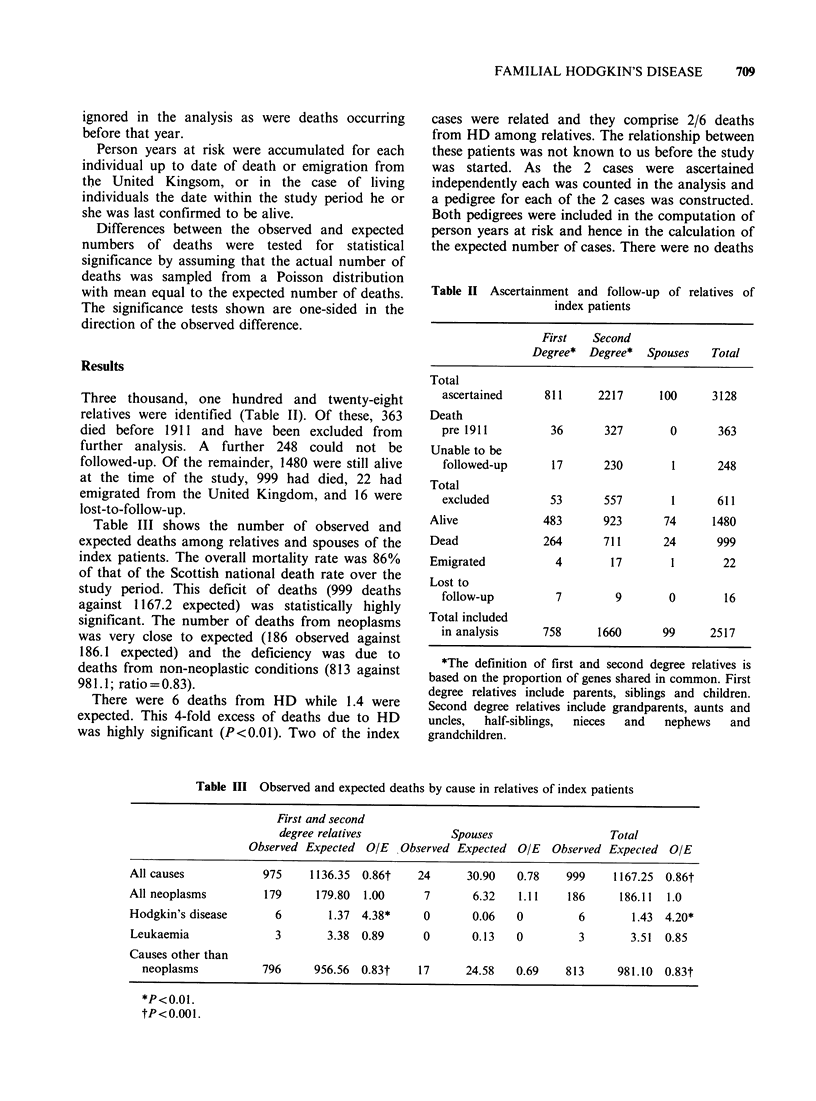

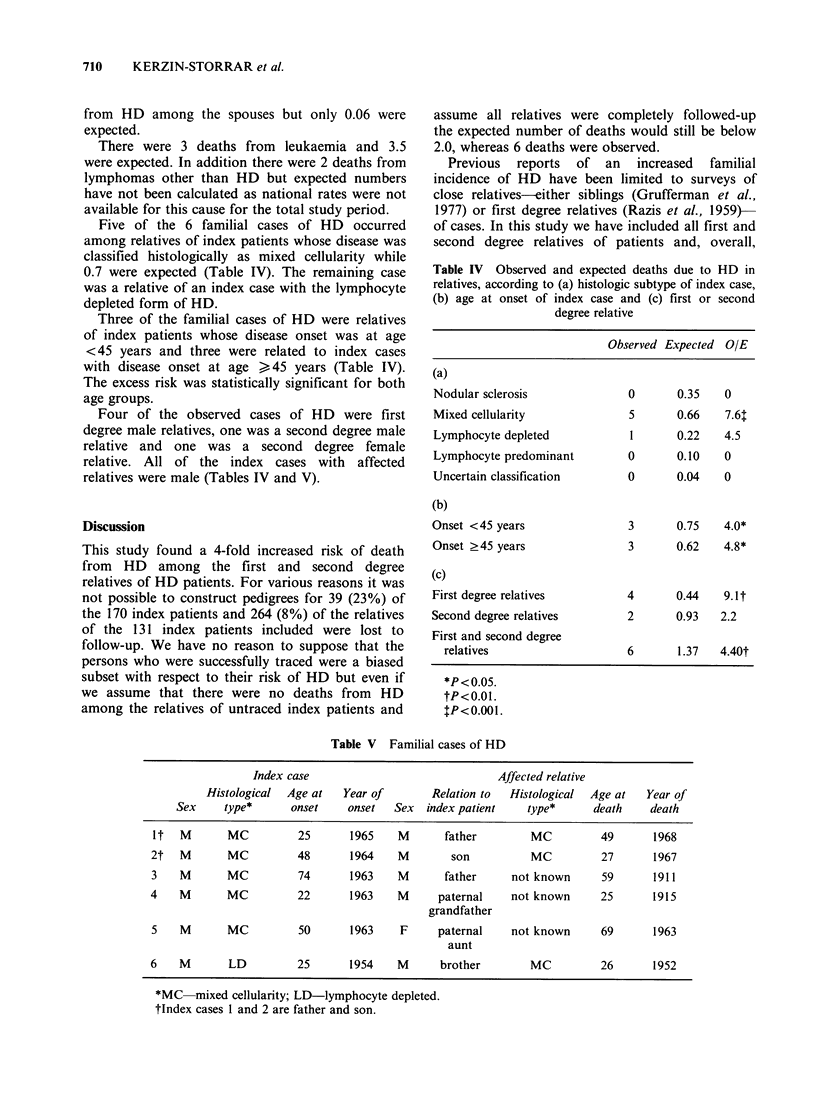

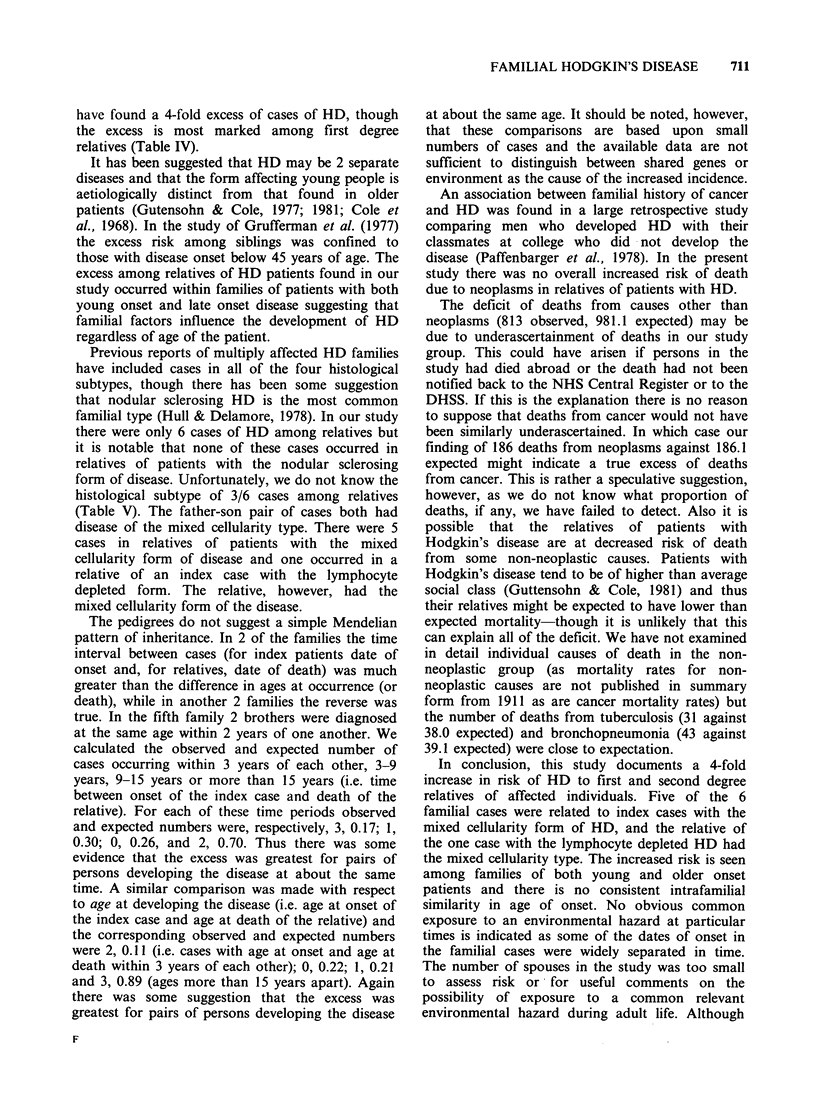

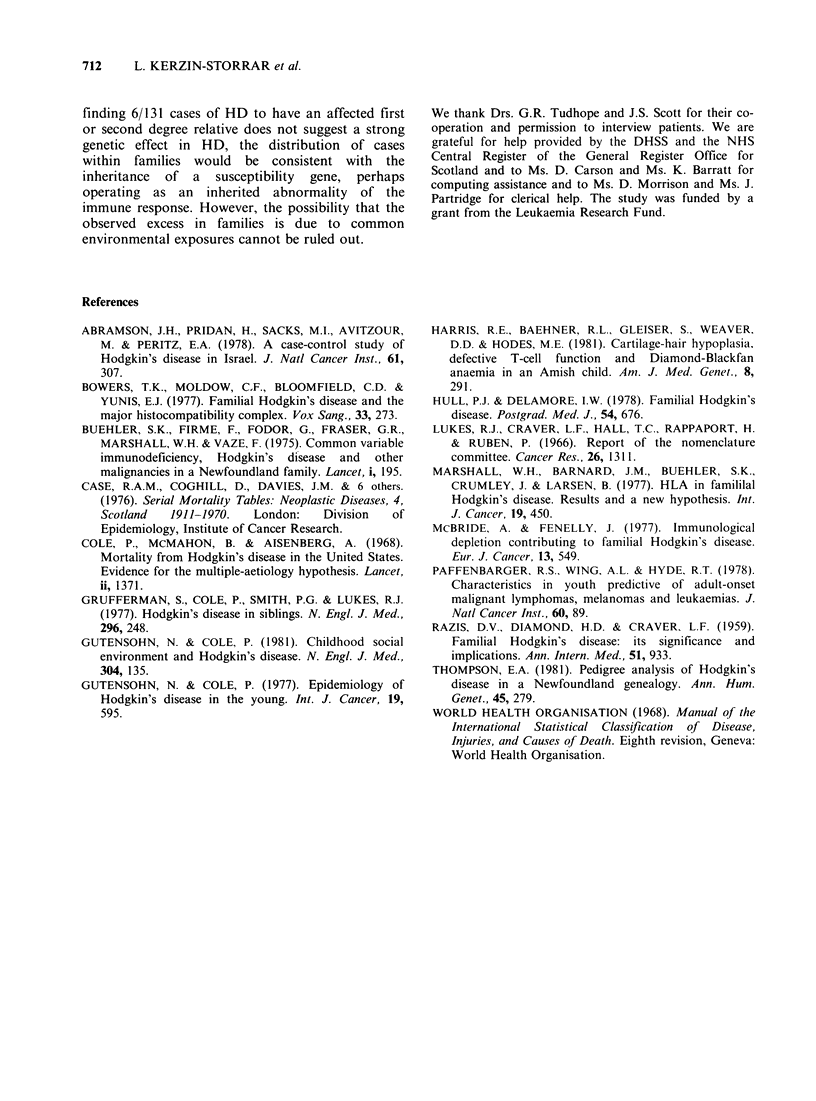


## References

[OCR_00595] Abramson J. H., Pridan H., Sacks M. I., Avitzour M., Peritz E. (1978). A case-control study of Hodgkin's disease in Israel.. J Natl Cancer Inst.

[OCR_00603] Bowers T. K., Moldow C. F., Bloomfield C. D., Yunis E. J. (1977). Familial Hodgkin's disease and the major histocompatibility complex.. Vox Sang.

[OCR_00606] Buehler S. K., Firme F., Fodor G., Fraser G. R., Marshall W. H., Vaze P. (1975). Common variable immunodeficiency, Hodgkin's disease, and other malignancies in a Newfoundland family.. Lancet.

[OCR_00617] Cole P., MacMahon B., Aisenberg A. (1968). Mortality from Hodgkin's disease in the United States. Evidence for the multiple-aetiology hypothesis.. Lancet.

[OCR_00623] Grufferman S., Cole P., Smith P. G., Lukes R. J. (1977). Hodgkin's disease in siblings.. N Engl J Med.

[OCR_00628] Gutensohn N., Cole P. (1981). Childhood social environment and Hodgkin's disease.. N Engl J Med.

[OCR_00633] Gutensohn N., Cole P. (1977). Epidemiology of hodgkin's disease in the young.. Int J Cancer.

[OCR_00638] Harris R. E., Baehner R. L., Gleiser S., Weaver D. D., Hodes M. E. (1981). Cartilage-hair hypoplasia, defective T-cell function, and Diamond-Blackfan anemia in an Amish child.. Am J Med Genet.

[OCR_00645] Hull P. J., Delamore I. W. (1978). Familial Hodgkin's disease.. Postgrad Med J.

[OCR_00654] Marshall W. H., Barnard J. M., Buehler S. K., Crumley J., Larsen B. (1977). HLA in familial Hodgkin's disease. Results and a new hypothesis;.. Int J Cancer.

[OCR_00660] McBride A., Fennelly J. J. (1977). Immunological depletion contributing to familial Hodgkin's disease.. Eur J Cancer.

[OCR_00665] Paffenbarger R. S., Wing A. L., Hyde R. T. (1978). Characteristics in youth predictive of adult-onset malignant lymphomas, melanomas, and leukemias: brief communication.. J Natl Cancer Inst.

[OCR_00671] RAZIS D. V., DIAMOND H. D., CRAVER L. F. (1959). Familial Hodgkin's disease: its significance and implications.. Ann Intern Med.

[OCR_00676] Thompson E. A. (1981). Pedigree analysis of Hodgkin's disease in a Newfoundland genealogy.. Ann Hum Genet.

